# Gemcitabine alone or in combination with cisplatin in patients with advanced or metastatic cholangiocarcinomas or other biliary tract tumours: a multicentre randomised phase II study – The UK ABC-01 Study

**DOI:** 10.1038/sj.bjc.6605211

**Published:** 2009-08-11

**Authors:** J W Valle, H Wasan, P Johnson, E Jones, L Dixon, R Swindell, S Baka, A Maraveyas, P Corrie, S Falk, S Gollins, F Lofts, L Evans, T Meyer, A Anthoney, T Iveson, M Highley, R Osborne, J Bridgewater

**Affiliations:** 1 Department of Medical Oncology, The Christie NHS Foundation Trust, Wilmslow Road, Manchester M20 4BX, UK; 2 Hammersmith Hospital, Du Cane Road, London W12 0HS, UK; 3 Institute of Cancer Studies, University of Birmingham, Vincent Drive, Birmingham B15 2TH, UK; 4 Hull Royal Infirmary, Princes Royal Hospital, Saltshouse Road, Hull HU8 9HE, UK; 5 Addenbrooke's Hospital, Hills Road, Cambridge CB2 2QQ, UK; 6 Bristol Haematology and Oncology Centre, Horfield Road, Bristol BS2 8ED, UK; 7 Ysbyty Glan Clwyd, Rhuddlan Road, Bodelwyddan, Rhyl LL18 5UJ, UK; 8 St George's Hospital, Blackshaw Road, London SW17 0QT, UK; 9 Weston Park Hospital, Sheffield S10 2SJ, UK; 10 Royal Free Hospital, Pond Street, London NW3 2QG, UK; 11 Cookridge Hospital, Cookridge, Leeds LS16 6QB, UK; 12 Southampton General Hospital, Tremona Road, Southampton SO16 6YD, UK; 13 Ninewells Hospital, Dundee, Scotland DD1 9SY, UK; 14 Dorset Cancer Centre, Poole Hospital, Longfleet Road, Poole, Dorset BH15 2JP, UK; 15 UCL Cancer Institute, 72 Huntley St, London WC1E 6AA, UK

**Keywords:** cholangiocarcinoma, gallbladder cancer, biliary tract, chemotherapy, gemcitabine, cisplatin

## Abstract

**Background:**

We assessed the activity of gemcitabine (G) and cisplatin/gemcitabine (C/G) in patients with locally advanced (LA) or metastatic (M) (advanced) biliary cancers (ABC) for whom there is no standard chemotherapy.

**Methods:**

Patients, aged ⩾18 years, with pathologically confirmed ABC, Karnofsky performance (KP) ⩾60, and adequate haematological, hepatic and renal function were randomised to G 1000 mg m^−2^ on D1, 8, 15 q28d (Arm A) or C 25 mg m^−2^ followed by G 1000 mg m^−2^ D1, 8 q21d (Arm B) for up to 6 months or disease progression.

**Results:**

In total, 86 patients (A/B, *n*=44/42) were randomised between February 2002 and May 2004. Median age (64/62.5 years), KP, primary tumour site, earlier surgery, indwelling biliary stent and disease stage (LA: 25/38%) are comparable between treatment arms. Grade 3–4 toxicity included (A/B, % patients) anaemia (4.5/2.4), leukopenia (6.8/4.8), neutropenia (13.6/14.3), thrombocytopenia (9.1/11.9), lethargy (9.1/28.6), nausea/vomiting (0/7.1) and anorexia (2.3/4.8). Responses (WHO criteria, % of evaluable patients: A *n*=31 *vs* B *n*=36): no CRs; PR 22.6 *vs* 27.8%; SD 35.5 *vs* 47.1% for a tumour control rate (CR+PR+SD) of 58.0 *vs* 75.0%. The median TTP and 6-month progression-free survival (PFS) (the primary end point) were greater in the C/G arm (4.0 *vs* 8.0 months and 45.5 *vs* 57.1% in arms A and B, respectively).

**Conclusion:**

Both regimens seem active in ABC. C/G is associated with an improved tumour control rate, TTP and 6-month PFS. The study has been extended (ABC-02 study) and powered to determine the effect on overall survival and the quality of life.

Biliary tract tumours are rare tumours accounting for 0.7% of malignant tumours in adults, with approximately 1200 new cases registered each year in England and Wales. The UK mortality rate is approximately 23 per million population with 1- and 5-year survival figures for adults diagnosed in England and Wales during 1986–1990 of 22 and 9%, respectively (Coleman *et al*, 1996).

Surgical resection, determined by the location, extent of disease and involvement of surrounding tissues, offer the only chance of long-term cure. Unfortunately, the resectability rates are generally low; survival after surgical resection varies widely between centres (range 23–50% at 5 years) (Klempnauer *et al*, 1997). Although this is better than the 5-year survival seen in pancreatic cancer (10% with surgery alone, increased to approximately 20% with adjuvant chemotherapy) (Neoptolemos *et al*, 2004; Oettle *et al*, 2007), the majority of patients with resected biliary tract cancers still develop recurrent or metastatic disease. In the presence of unresectable disease, patients with biliary tumours often present with the additional clinical problem of biliary obstruction. Patients need to be adequately palliated before further treatment (e.g. chemotherapy) either by endoscopic or percutaneous biliary stenting.

Most patients present with tumours that are too advanced for surgical resection, and the function of radiotherapy or chemotherapy at present remains uncertain. Before initiating this study, a review of the chemotherapy regimens used for the treatment of biliary tract cancers showed that the majority of chemotherapy regimens used to date have been 5-FU based (Smith *et al*, 1984; Kajanti and Pyrhonen, 1994; Comella *et al*, 1996; Gebbia *et al*, 1996; Patt *et al*, 1996, 1999; Ducreux *et al*, 1998; Raderer *et al*, 1999; Choi *et al*, 2000). As a single agent, intravenous 5-FU has a response rate of 25–32% (Raderer *et al*, 1999; Choi *et al*, 2000). The addition of cisplatin (Ducreux *et al*, 1998; Patt *et al*, 1999), doxorubicin (Patt *et al*, 1999), epirubicin (Kajanti and Pyrhonen, 1994), hydroxyurea (Gebbia *et al*, 1996) or methotrexate (Kajanti and Pyrhonen, 1994; Comella *et al*, 1996) seems to add little in terms of response rate in these phase II studies. The oral fluoropyrimidine pro-drug, capecitabine, has shown promising results (Lozano *et al*, 2000) in 26 patients with cholangiocarcinomas and gallbladder cancers, but confirmation of efficacy in a randomised phase III study is not available.

Docetaxel has been found to be active and well tolerated on the basis of a 25% RR and 31% stabilisation of disease in a mixed group of gallbladder (*n*=13), cholangiocarcinomas (*n*=4) and ampullary tumours (*n*=3) (Agelaki *et al*, 1999).

The adoption of gemcitabine as the standard of care for patients with pancreatic cancer (Casper *et al*, 1994; Rothenberg *et al*, 1996; Burris *et al*, 1997; Storniolo *et al*, 1997) led to interest in the use of gemcitabine for other hepatobiliary tumours. In the usual dosing regimen, responses of 16–42% have been obtained (Raderer *et al*, 1999; Gallardo *et al*, 2000). Some investigators have looked at altering the schedule to improve efficacy; Dragovich *et al* (2000) found that the fixed dose rate (at 10 mg m^−2^ min^−1^) did not enhance efficacy and that the response rates with intra-arterial administration are not significantly higher than intravenous historical controls (Weissmann and Ludwig, 1999). Initial data from a continuous-infusion regimen showed this to be well tolerated and activity data were awaited (Eckel *et al*, 2000). The median survivals reported in these early phase II studies ranged from 5.7 to 11+ months.

Cisplatin is widely used in combination chemotherapy and there are synergistic effects when it is combined with gemcitabine. On account of this, the combination of cisplatin and gemcitabine is widely used in other cancers (including lung, head and neck, pancreatic and bladder cancers). The specific sequence of cisplatin followed by gemcitabine seems to be optimal in pre-clinical testing (Braakhuis *et al*, 1995; Theodossiou *et al*, 1998). To exploit this additive/synergistic effect, we used a regimen that delivered both agents at every dosing time point. Our group has previously shown this regimen to be well tolerated and easily deliverable as a 2-h out-patient infusion regimen in patients with pancreatic cancer. Although the dose of cisplatin seems low (25 mg m^−2^), it equates to a dose of 50 mg m^−2^ per 21-day cycle, and earlier attempts at increasing the dose intensity resulted in more dose delays and interruptions (Clayton *et al*, 2006).

The aim of this study was to evaluate both single-agent gemcitabine and the cisplatin/gemcitabine doublet in patients with locally advanced or metastatic biliary tract tumours (cholangiocarcinomas and gallbladder carcinomas) using a randomised phase II design. A follow-on phase III trial was also planned depending on the relative merits of each treatment arm in terms of activity, feasibility and tolerability.

The primary objective was to assess the efficacy in terms of 6-month progression-free survival (PFS) for both treatment arms. Secondary objectives included response rate, overall survival and toxicity assessment.

## Materials and methods

### Study design

**Figure 1:**
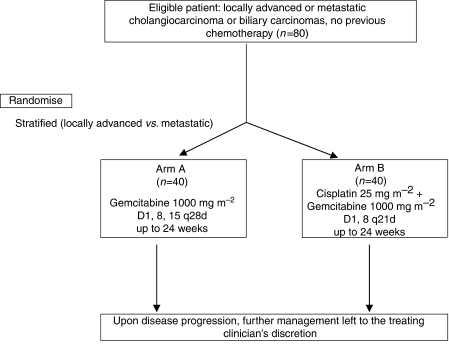
Trial schema.

This is a multicentre randomised phase II study of weekly (for 3 weeks in every four-week cycle, × 6 cycles) doses of gemcitabine 1000 mg m^−2^ as a single agent or preceded by cisplatin 25 mg m^−2^ (on a 2 weeks in every three-week cycle, × 8 cycles) in patients with histologically or cytologically verified, non-resectable or recurrent/metastatic cholangiocarcinoma (intra- or extrahepatic), gallbladder or ampullary carcinoma (Figure 1).

Eligibility criteria included the following: no earlier chemotherapy or radiotherapy, Karnofsky performance (KP) status ⩾60, age ⩾18 years, predicted survival ⩾12 weeks, adequate bone marrow, liver, renal and cardiac function, adequate biliary drainage, no known brain metastases, no previous malignancy, no serious concurrent medical illness and, where applicable, approved methods of birth control. All patients gave written informed consent and the trial was conducted in accordance with ICH-GCP standards and the Declaration of Helsinki. Each participating institution was required to have approval by their respective local research ethics committee.

### Treatment

Patients were randomised to receive gemcitabine 1000 mg m^−2^ intravenous infusions on days 1, 8 and 15 of each 28-day cycle or cisplatin 25 mg m^−2^ followed by gemcitabine 1000 mg m^−2^ by intravenous infusions on days 1 and 8 of each 21-day cycle.

A minimum of two cycles was required to assess tumour status and the maximum period of therapy was 24 weeks (six cycles of single-agent gemcitabine and eight cycles of cisplatin/gemcitabine).

Patients were assessed after every cycle for adverse events; toxicities were graded according to the Revised Common Toxicity grading Criteria version 2.0. A complete blood count, biochemistry, physical examination and urine analysis were also assessed at the commencement of each cycle. Radiological assessment by CT scan every 12 weeks during treatment determined the tumour status. Objective tumour response was evaluated according to the WHO criteria by local investigators; central radiology review was not performed.

Treatment was given until progressive disease (or until completion of the planned 24 weeks of therapy), unacceptable toxicity or patient refusal. Thereafter, patients continued to be followed up for survival data. Patients who had not progressed by the end of the treatment period continued to have CT scans at 3-month intervals until such time as there was evidence of disease progression.

### Statistical design

These are both relatively new regimens for this type of tumour. This randomised phase II study aimed at assessing the relative merits of both regimens before proceeding to a full phase III study.

The trial consisted of 80 patients, randomised 1 : 1 between the two regimens. Patients were stratified, at randomisation, for the extent of disease (locally advanced *vs* metastatic). Given the difficulties in measuring the response rate in this group of patients (where often the disease is concentric around a bile duct), the primary end point was PFS at 6 months from the time of randomisation. Assuming a baseline 40% PFS at 6 months for the gemcitabine arm, the cisplatin/gemcitabine combination arm would be considered ‘favourable’ if there was a 10% improvement in this end point (to 50%) with an acceptable toxicity profile. A total of 40 patients in each arm would estimate this 6-month progression-free rate with an accuracy of ±15.5%.

This study was not powered to permit formal statistical comparison between the two treatment arms. However, it would allow an initial assessment of the regimens in terms of a 6-month progression-free rate, response rate, overall survival and toxicity with a view to a follow-on phase III study.

## Results

**Figure 2:**
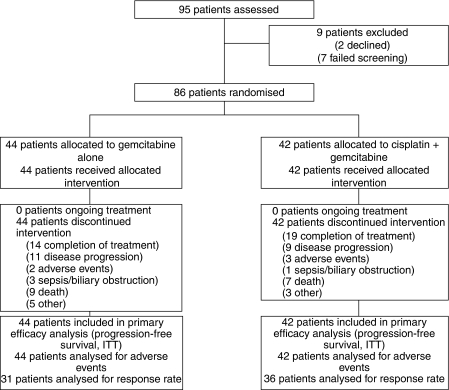
Trial profile.

From February 2002 to May 2004, 86 patients (median age 63 years, range 29–84 years with a slight preponderance of women) were randomised from 15 institutions, 44 to the gemcitabine arm and 42 to the cisplatin/gemcitabine combination (Figure 2). Baseline characteristics including median age, sex, performance score (KP), primary tumour site (cholangiocarcinoma, gallbladder or ampullary cancer), earlier surgery, indwelling biliary stent and disease stage (locally advanced *vs* metastatic) are comparable between the treatment arms (Table 1).

### Toxicity

Both treatments were well tolerated and the toxicities were much as expected from the experience in other tumours (Table 2). In keeping with the nature of biliary tract cancers, non-neutropenic infections were fairly common at some point during the treatment (mostly cholangitis with biliary obstruction), occurring with similar frequency in both arms and usually requiring admission to hospital for biliary drainage and antibiotic therapy. The most frequently reported (>10% incidence) grade 3–4 drug-related adverse events on the single gemcitabine arm were transaminitis (13.6%) and neutropenia (also 13.6%), whereas in the combination arm, lethargy, neutropenia, thrombocytopenia and transaminitis occurred in 28.6, 14.3, 11.9 and 11.9% of cases, respectively. Although the incidence of lethargy was higher in the combination arm (28.6 *vs* 9.1% in the gemcitabine-alone arm), this did not result in an increase in withdrawal from treatment (*n*=3 in combination arm *vs n*=2 in gemcitabine-alone arm), Figure 2.

### Response

A total of 67 patients were evaluable for tumour response in accordance with the protocol and WHO tumour response criteria, 31 on the gemcitabine arm and 36 on the cisplatin/gemcitabine arm. No complete tumour responses were observed. In total, 7 patients on the gemcitabine arm had a partial response compared with 10 patients on the cisplatin/gemcitabine arm (PR 22.6 *vs* 27.8%). In addition, 11 patients had stable disease on gemcitabine alone *vs* 17 on the combination (SD 35.5 *vs* 47.2%) The tumour control rate (CR+PR+SD) was 58.0 *vs* 75.0% in favour of the cisplatin/gemcitabine combination. In total, 13 and 9 patients, respectively, had progressive disease (Table 3).

Patients were required to discontinue therapy on radiological evidence of disease progression or unacceptable toxicity. Table 4 shows that the mean duration of time on treatment was longer for patients receiving cisplatin/gemcitabine *vs* gemcitabine alone (18.7 *vs* 15.7 weeks, respectively).

### Survival

**Figure 3:**
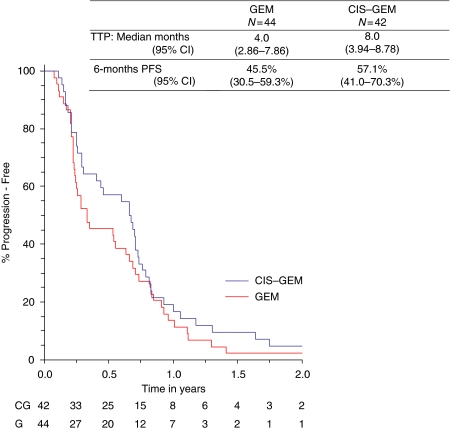
Kaplan–Meier curve of progression-free survival by treatment arm as a function of time.

The primary end point for this study was 6-month PFS. At the time of analysis, all patients had progressed. The 6-month PFS for the gemcitabine-alone arm was 45.5% (95% CI 30.5–59.3%) *vs* 57.1% (95% CI 41.0–70.3%) for the combination arm with median PFSs of 4.0 and 8.0 months, respectively, in each of the arms (Figure 3). Overall survival data have been censored by the Data Safety Monitoring Committee, as the study is not powered to allow a comparison between the arms in terms of survival and all patients in this study will be included in the survival statistics in the follow-on phase III study (statistically powered for survival).

## Discussion

Surgical resection remains the treatment of choice for patients with biliary tract cancers and, to date, there are no conclusive data to support the routine use of chemotherapy in patients diagnosed with locally advanced, inoperable, recurrent or metastatic disease.

A number of limitations have contributed to this lack of evidence base including the relative infrequency of these cancers, particularly in Western countries, with any individual institution having small numbers of patients. A recent review of all published (and abstract) biliary tract cancer trials revealed that the numbers of patients per trial ranged from 5 to 65 (with a mean of 25.1 patients) (Eckel and Schmid, 2007). Our ABC-01 study, with 86 patients is, therefore, the largest study reported in the literature to date, enabled by a national, UK-wide National Cancer Research Network (NCRN) collaboration.

Another limitation is that biliary tract tumours are notoriously difficult to evaluate for response or progression. Although some patients have parenchymal lesions that lend themselves to objective measurement, many patients have malignant sclerosing disease along bile ducts, which, even in the presence of a response, may show little change on conventional imaging. In total, 19 of our 86 patients (22%) did not have evaluable disease. In their pooled analysis, Eckel and Schmid have shown that the response rate correlates poorly with overall survival (correlation coefficient *r*=0.2, *P*=0.043). Tumour control rate fares a little better (*r*=0.26, *P*=0.024), although time to tumour progression seems the best surrogate marker of activity in this disease group with the best correlation with overall survival (*r*=0.73, *P*<0.0005) (Eckel and Schmid, 2007). This finding validates the primary end point chosen for our study, which was based on radiological or clinical progression of disease. Our observation of improved 6-month PFS from 45.5% (gemcitabine alone) to 57.1% (cisplatin/gemcitabine combination) was close to our approximated statistical assumption of 10% benefit on which our cohort size was based. Moreover, patients who received gemcitabine alone fared a little better than we had assumed (initial assumption of 6-month PFS: 40%). Given the limitations of conventional cross-sectional imaging, the use of functional imaging (with, for example, FDG-PET or FLT-PET) may provide more reliable surrogates of activity to hasten drug development for the treatment of biliary tract cancers (as is currently being investigated for other tumour types, e.g. colorectal cancer (Francis *et al*, 2004)).

Thus, both treatment arms in ABC-01 showed activity. However, combination chemotherapy resulted in a marginally better response rate (CR+PR: 27.8 *vs* 22.6%), and superior tumour control rate (CR+PR+SD: 75.0 *vs* 58.0%), broadly in line with that seen in previous studies (Dingle *et al*, 2005). Both treatment arms were well tolerated, although patients who received doublet therapy showed a significant increase in lethargy (28.6 *vs* 9.1%); the effect of this finding on the quality of life was not explored in this study. Neither did we mandate serum CA 19-9 measurement, as this was not widely available as a standard test at the outset of the study.

The aforementioned review found that most of the studies in the literature were small phase II studies evaluating mostly fluoropyrimidine- and gemcitabine-based therapies (others include taxanes, anthracyclines, mitomycin-C and irinotecan) (Eckel and Schmid, 2007) and two other randomised phase II studies have been reported in the literature: an EORTC study comparing high-dose 5-FU with or without cisplatin (Ducreux *et al*, 2005) and another study comparing mitomycin-c with gemcitabine *vs* mitomycin-C and capecitabine (Kornek *et al*, 2004).

There is a paucity of randomised phase III data available. In one phase III study, 90 patients with both pancreatic (*n*=53) and biliary tumours (*n*=37) were randomised to receive either best supportive care (BSC) or BSC with 5-FU, etoposide and leucovorin (FELV) chemotherapy (etoposide was omitted for elderly patients because of the high incidence of mucositis). There was a statistically significant survival advantage from the chemotherapy arm in terms of survival (6 *vs* 2.5 months, *P*<0.01, despite a crossover to treatment of eight patients allocated to the BSC arm), although the study was underpowered to make any conclusion regarding biliary tract cancers (Glimelius *et al*, 1996). A more recent study by Rao *et al* (2005) built on the findings of this study by randomising 54 patients to either FELV chemotherapy or to the epirubicin, cisplatin and continuous-infusion 5-FU (ECF) regimen. The authors found the ECF regimen to be less toxic than FELV with a similar response rate, symptom resolution and failure-free survival, although it did not improve survival. The study, which had planned to recruit 166 patients, closed because of slow accrual, showing the difficulty of large studies in this patient group.

ABC-01, although not powered to permit a formal comparison between the two treatment arms, has provided useful information in terms of 6-month progression-free rate, response rate, overall survival and toxicity. It has been followed by ABC-02, a randomised phase III study, also run under the auspices of the UK-NCRN, comparing the same two treatment arms but powered to detect a survival advantage as the primary end point. In addition, the effect of either treatment on the quality of life and changes in serum CA19-9 will be reported.

## Figures and Tables

**Table 1:** Patient and tumour characteristics

	**GEM**	**CIS/GEM**	**Total**
No. of patients	44	42	86
Male	19 (43.2%)	17 (40.5%)	36 (41.9%)
Female	25 (56.8%)	25 (59.5%)	50 (58.1%)
Median age, years (range)	64 (29–84)	63 (38–76)	63 (29–84)
			
*Primary tumour site*			
Intrahepatic CC	7 (15.9%)	12 (28.6%)	19 (22.1%)
Extrahepatic CC	11 (25.0%)	9 (21.4%)	20 (23.3%)
Cholangiocarcinoma NOS	10 (22.7%)	10 (23.8%)	20 (23.3%)
Gallbladder	12 (27.3%)	10 (23.8%)	22 (25.6%)
Ampullary cancer	4 (9.1%)	1 (2.4%)	5 (5.8%)
			
*Stage*			
Locally advanced	11 (25.0%)	16 (38.1%)	27 (31.4%)
Metastatic	33 (75.0%)	26 (61.9%)	59 (68.6%)
			
*Earlier therapy*			
Curative surgery	10 (22.7%)	3 (7.1%)	13 (15.1%)
Palliative surgery	10 (22.7%)	13 (31.0%)	23 (26.7%)
Laparotomy only	17 (38.6%)	12 (28.6%)	29 (33.7%)
Biliary stent insertion	23 (52.3%)	25 (59.5%)	48 (55.8%)
Radiotherapy	3 (6.8%)	0 (0.0%)	3 (3.5%)
			
*Karnofsky performance score*			
100	4 (9.1%)	5 (11.9%)	9 (10.5%)
90	17 (38.6%)	18 (42.9%)	35 (40.7%)
80	19 (43.2%)	12 (28.6%)	31 (36.0%)
70	2 (4.5%)	6 (14.3%)	8 (9.3%)
60	1 (2.3%)	1 (2.4%)	2 (2.3%)
NR	1 (2.3%)	0 (0.0%)	1 (1.2%)

CC=cholangiocarcinoma; CIS/GEM=cisplatin/gemcitabine; NOS=not otherwise specified; NR=not recorded.

**Table 2:** Grade 3–4 toxicity by treatment arm (given as incidence of toxicity by patient)

	**GEM**	**CIS/GEM**
**Toxicity**	**No. of patients *n*=44**	**No. of patients *n*=42**
*Haematological adverse events*		
Anaemia	2 (4.5%)	1 (2.4%)
Leucopenia	3 (6.8%)	2 (4.8%)
Neutropenia	6 (13.6%)	6 (14.3%)
Thrombocytopenia	4 (9.1%)	5 (11.9%)
		
*Non-haematological adverse events*		
Lethargy	4 (9.1%)	12 (28.6%)
Infection (non-neutropenic)	7 (15.9%)	8 (19.0%)
Bilirubin	9 (20.5%)	5 (11.9%)
Transaminases	6 (13.6%)	5 (11.9%)
Vomiting	0 (0.0%)	3 (7.1%)
Oedema	2 (4.5%)	2 (4.8%)
Anorexia	1 (2.3%)	2 (4.8%)
Pain	1 (2.3%)	2 (4.8%)
Diarrhoea	0 (0.0%)	2 (4.8%)
Dyspnoea	0 (0.0%)	2 (4.8%)
Constipation	1 (2.3%)	1 (2.4%)
Infection (neutropenic)	0 (0.0%)	1 (2.4%)
Stomatitis	0 (0.0%)	1 (2.4%)
Renal	0 (0.0%)	1 (2.4%)
Neuropathy	1 (2.3%)	0 (0.0%)
Nausea	0 (0.0%)	0 (0.0%)
Alopecia	0 (0.0%)	0 (0.0%)

CIS/GEM=cisplatin/gemcitabine.

**Table 3:** Radiological response to treatment (WHO criteria, for evaluable patients only)

	GEM *N*=31*	CIS/GEM *N*=36*
Not evaluable^*^	13/44 (29.5%)	6/42 (14.3%)
Complete response (CR)	0	0
Partial response (PR)	7 (22.6%)	10 (27.8%)
Stable disease (SD)	11 (35.5%)	17 (47.2%)
Progressive disease (PD)	13 (41.9%)	9 (25.0%)
Response rate (CR+PR)	7 (22.6%)	10 (27.8%)
Tumour control rate (CR+PR+SD)	18 (58.0%)	27 (75.0%)
Not assessable	13 (25.0%)	6 (11.9%)

CIS/GEM=cisplatin/gemcitabine. ^*^Patients were not required to have measurable disease at study entry.

**Table 4:** Delivery of therapy by treatment arm

	**Gemcitabine *n*=44**	**Cisplatin/gemcitabine *n*=42**
No of cycles given	158	246
Duration of cycle	4 weeks	3 weeks
Mean duration of treatment (weeks)	15.7	18.7
Median number of cycles	3	7.5
Range	1–6	1–8
